# Investigating the Skill Development of Medical Students in Focused Assessment With Sonography for Trauma (FAST) Ultrasound: A Comparative Analysis Across Different Stages of Medical Training

**DOI:** 10.7759/cureus.44414

**Published:** 2023-08-30

**Authors:** Michael Atalla, Andrew Yacoub, Hasan Al-Ali, Bianca Lupia, Layal Ezzeddine, Shaliz Barzani, Michelle Moussa, James Coey, Tarek Alambrouk, Haider Hilal

**Affiliations:** 1 School of Medicine, St. George's University School of Medicine, True Blue, GRD; 2 Medical Imaging, University of Toronto, Toronto, CAN; 3 Faculty of Science, University of Waterloo, Waterloo, CAN; 4 Anatomy, St. George's University School of Medicine, Newcastle upon Tyne, GBR; 5 School of Medicine, St. George's University School of Medicine, Newcastle upon Tyne, GRD; 6 School of Medicine, St. George's University School of Medicine, Newcastle upon Tyne, GBR

**Keywords:** medical education, medical student assessment, medical student training, ultrasound-guided, ultrasound imaging

## Abstract

Introduction: Focused assessment with sonography for trauma (FAST) ultrasound (US) is a valuable medical examination used in trauma settings, particularly for rapid responses to events such as natural disasters. Although the efficacy and benefits of FAST in patient care have been extensively studied, there is limited research on training medical students in FAST. Previous studies have found that medical students can proficiently perform a FAST US after two days of training. However, these studies exclusively included first-year medical students without considering variations in their medical knowledge. Particularly, the advantage of medical students having US experience before undergoing FAST training has not been previously examined.

Objectives: Assess the performance and knowledge acquisition of medical students with and without prior US experience after completing a FAST training course.

Methods: The study included a total of 71 students, consisting of 33 males and 38 females, who were between the ages of 18 and 31, with an average age of 24.6 and a standard deviation of 2.4. The inclusion criteria targeted first- and second-year medical school students who participated on a volunteer basis. Students were divided into two groups: group A, consisting of those without prior US experience, and group B, made up of those who had previous US experience. All students completed a pre-training survey to share their comfort and confidence in US use and knowledge. A baseline FAST exam was conducted to establish initial performance. A comprehensive three-hour training session was then provided. Post-training, students performed another FAST exam to assess improvement, followed by a post-training survey to evaluate comfort and confidence.

Results: Medical students who had prior experience in the US (group B) performed significantly better (p<0.01) in both the pre- and post-training FAST exams when compared to students without previous US experience. Specifically, in locating the liver, right kidney, hepatorenal recess, and left kidney, as well as detecting fluid accumulation when in a supine position. Additionally, medical students with prior US experience (group B) exhibited higher baseline confidence (p<0.005-p<0.01) in their ability to perform a FAST exam, as indicated by the results of the pre-testing survey.

Conclusion: Previous experience with US significantly boosted confidence and knowledge gains following FAST training. This emphasizes the value of including US training in medical school programs after earlier exposure, offering evident benefits. The study reveals the unexplored benefit of having prior US experience for medical students undergoing FAST training, thus addressing a previously unexplored area in current research. The conclusions stress the necessity of integrating US training into medical school curricula after initial exposure. This understanding can direct medical educators in refining the education process, enabling students to be better equipped for real-world medical situations involving FAST.

## Introduction

The escalating utilization of ultrasound (US) in the medical realm can be attributed to its bedside accessibility and non-invasive nature, with the focused assessment with sonography in trauma (FAST) technique exemplifying its potential [1]. This trend is further exemplified by point-of-care US (POCUS), extended-focused assessment with sonography in trauma (e-FAST), and evaluations of cardiac function and pulmonary conditions through US [2,3]. FAST, primarily designed to assess hemoperitoneum and hemopericardium, has expanded to e-FAST, encompassing pleural spaces for pneumothorax and hemothorax. POCUS, meanwhile, serves as a valuable tool for cardiac and lung evaluations [2,3]. The urgency behind emphasizing the FAST technique arises from the global prevalence of abdominal trauma, which ranks third in trauma-related mortality after traumatic brain and chest injuries [4]. Rapid identification of abdominal trauma through FAST holds a significant potential for mitigating needless trauma-induced fatalities. Importantly, these diagnostic methods have become the standard in emergency departments globally, providing non-surgical avenues for trauma case assessments [1].

In light of this evolving landscape, the incorporation of US education into medical school curricula assumes pivotal significance. Equipping medical students with skills in ultrasonography during their pre-clinical years emerges as a pressing necessity to expedite its assimilation within the medical framework. This approach not only ensures prompt and effective care for post-trauma patients but also offers comprehensive training to students before embarking on their clinical journey. Notably, existing studies underscore the transformative impact of US training, enhancing students' competence, enthusiasm for US examinations, and anatomical comprehension [5,6].

Despite the recognized advantages of integrating US instruction into medical school curricula, a substantial gap in research remains regarding the optimal timing for introducing US training to medical students [7]. The core "need" driving this study is to ascertain whether students should commence FAST US training early in their pre-clinical years or after gaining foundational familiarity with US during later pre-clinical training. This critical question underscores the influence of training timing on student proficiency and forms the crux of this investigation.

St. George's University (SGU) School of Medicine, renowned for its diverse medical programs, offers standard four-year tracks and extended five- or six-year tracks. The latter includes preparatory years of pre-medical courses prior to the core four-year medical curriculum. Remarkably, while ultrasound education is seamlessly integrated into the four-year curriculum, it remains conspicuously absent during the preparatory phase of the five-year track. The study's focus remains entrenched in students during their preparatory year and the initial year of the medical curriculum within the five-year track [8].

The study's ultimate objective is to comprehensively evaluate the proficiency of first-year students in both cohorts (groups A and B) as they master the FAST examination. This evaluation encompasses their capacity to adeptly diagnose patients, elevate diagnostic accuracy, and minimize the need for supplementary imaging studies. By meticulously comparing students' performance in mastering the FAST examination across distinct stages of clinical training, this study lays the foundation for the seamless integration and effective utilization of US within their medical careers, underscoring the profound "need" for such research.

## Materials and methods

Study design

A FAST US training session took place at St. George’s University (SGU), Northumbria Campus (NU) in the United Kingdom. This mixed methods study incorporated both survey and assessment components and included medical students with and without prior US training experience. At SGU/NU School of Medicine, US training is integrated into the five-year curriculum, starting in the second and third years, whereas first-year students do not receive any US training. A total of 71 students, with ages ranging from 18 to 31 (average age: 24.6, standard deviation: ±2.4) and consisting of 33 males and 38 females, were included in the study. The participants were first- and second-year medical school students who volunteered for the study. Group A consisted of first-year medical students without any prior US training, while group B comprised second-year medical students who had undergone at least four sessions of previous US training. Over a 21-day period, from March 16 to April 6, 2023, the students received FAST training and underwent assessment in four sessions. There was financial compensation; however, participants received a certificate upon completion of the FAST US workshop, which supplemented their ongoing course of study. All participants were 18 years of age or older and provided voluntary, informed consent to partake in the study.

Intervention

Students assigned to group A had no prior experience with US training, although they possessed a foundational knowledge of the organs evaluated and examined during the FAST training session. These students in the five-year program, classified as pre-medical students, had received instruction in the basic sciences. In contrast, group B students had previous exposure to path US techniques and demonstrated a strong foundational understanding of the basic sciences.

**Figure 1:**
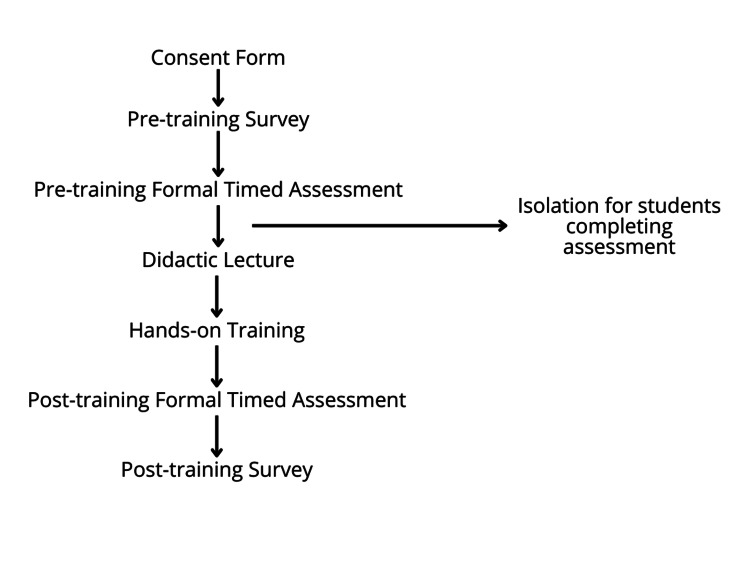
Flow chart of seven-part US training workshop. US: ultrasound.

**Figure 2:**
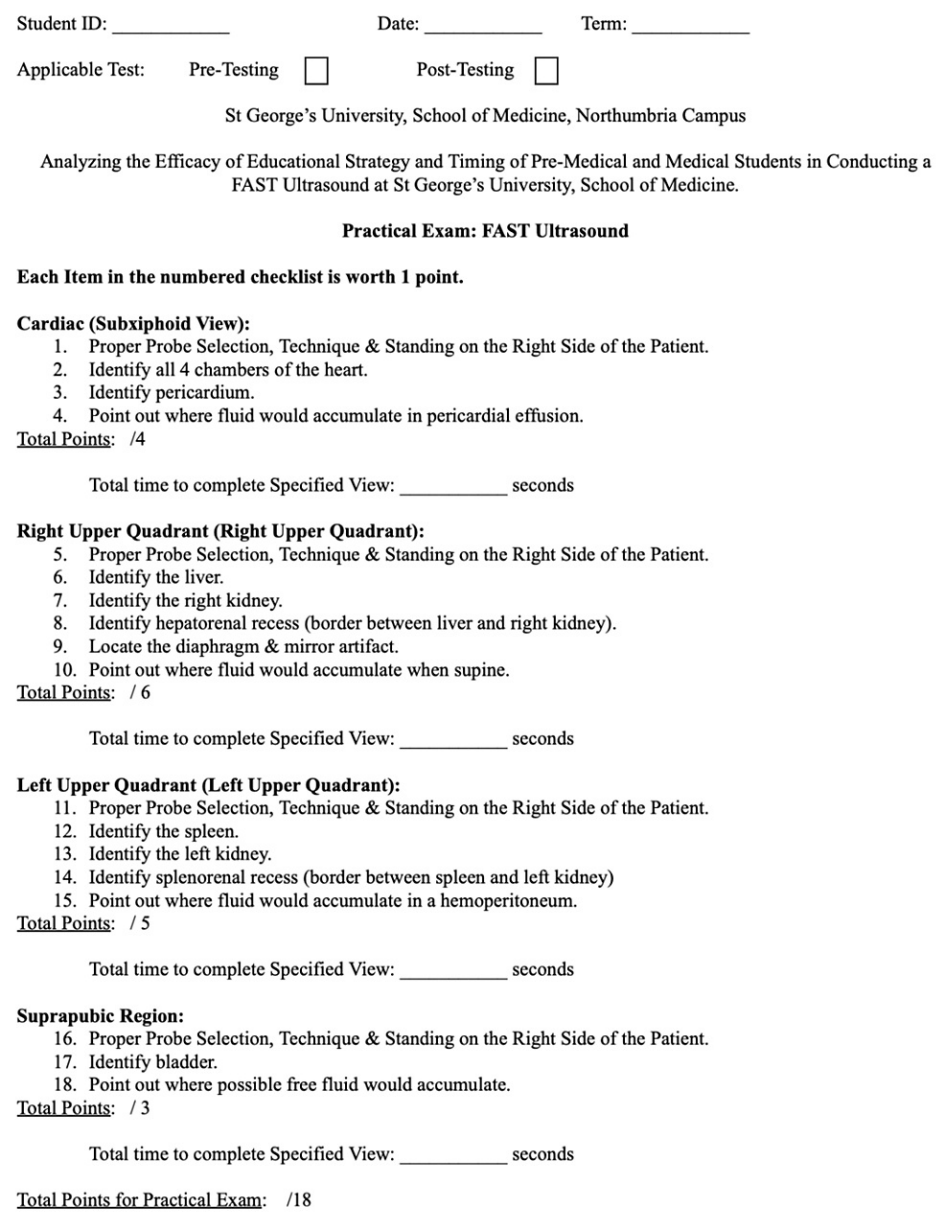
Assessment exam for FAST US: pre- and post-training. FAST: focused assessment with sonography for trauma, US: ultrasound.

**Figure 3:**
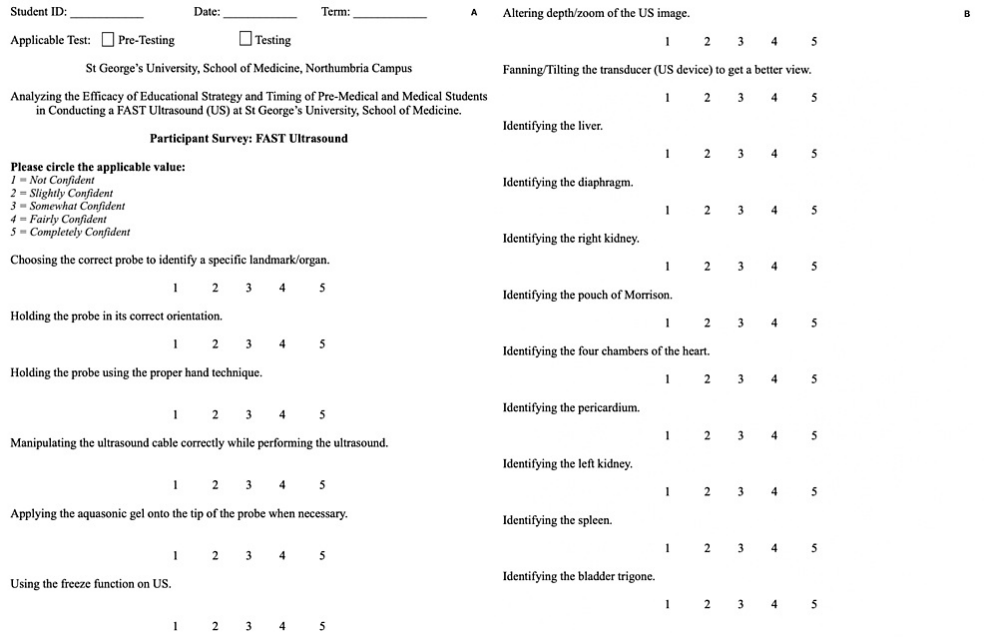
Survey for FAST US: pre- and post-training. FAST: focused assessment with sonography for trauma, US: ultrasound.

The participants of this study underwent a comprehensive seven-part US training workshop, which encompassed a pre-training survey, a pre-training formal timed assessment, a didactic lecture, hands-on training, a post-training formal timed assessment, and a post-training survey (Figure 1). The training program commenced after the completion of a formal timed assessment (Figure 2) and a pre-training survey (Figure 3), which served to establish a baseline and ascertain the students' pre-existing US proficiency and skills. Subsequently, the students received instruction in a didactic workshop, which focused on the essential components of a US machine, the proper conduct of ultrasonography, and the specific objectives associated with FAST as performed by medical professionals. The didactic workshop primarily emphasized instructing students on the four main views integral to FAST US: the right upper quadrant, left upper quadrant, cardiac (subxiphoid pericardial window), and suprapubic region. Before any US training, a formal timed assessment was administered to gauge the students' initial US proficiency and to serve as a comparative reference for participants.

Following the didactic component, students were trained in the execution of FAST. They received a step-by-step demonstration on operating the US machine and actively participated in a hands-on training seminar, during which they practiced conducting FAST examinations on volunteer patients. To evaluate the efficacy of the training, a post-training formal timed assessment (Figure 2) was conducted, and a written post-test survey (Figure 3) was completed at the conclusion of the final session, allowing for a comparison with the pre-test survey. The same survey and formal timed assessment rubric were used for both the pre-training and post-training components to ensure consistency and reliability of the evaluation process. Additionally, consistent assessors and facilitators were assigned to the four sessions at every station to mitigate the potential for inter-session bias between the two groups. ​​Students who were completing the pre-training assessments were isolated from the remaining students to prevent bias.

Ultrasound curriculum

The students underwent training in using the abdominal probe of a LOGIQ™ 𝘦 cart-based US machine (GE Healthcare, Chicago, IL) in order to conduct the FAST examination. A proficient instructor from the faculty of SGU/NU provided a comprehensive demonstration of the appropriate US techniques, skills, and image acquisition procedures. Prior to their final formal assessment, the students independently practiced acquiring accurate images with the assistance and guidance of a trained instructor.

The workshop provided instruction on the use of three distinct cart-based LOGIQ™ 𝘦 machines. Among these machines, one was specifically designated for the formal demonstration, which was skilfully conducted by a trained researcher. The remaining two LOGIQ™ 𝘦 cart-based machines were exclusively employed for the purpose of conducting formal examinations of the participating students.

Data collection

The workshop commenced with a survey administered to the participants to gauge their previous experience with US, and their level of comfort in completing each section of a FAST prior to any training. The survey utilized a Likert scale ranging from 1 to 5, where 1 represented "strongly disagree" and 5 denoted "strongly agree."

The pre-training formal timed assessment encompassed a four-part comprehensive FAST scan of a volunteer patient. Each section evaluated the students' probe selection, technique, and time taken to complete the section. The student's performance was assessed in real-time by trained examiners on an individual basis. The grading system ranged from 0 to 1, based on the student's technique and the quality of the US image obtained. An image was deemed insufficient (0 point) if it was not clear or if the participant failed to identify the specified structure. Conversely, a sufficient (1 point) grade indicated a clear image and successful identification of the specified structure. If a student failed to identify all structures within the allotted time frame, they received a mark of 0 for the unidentified structures. The maximum score a student could achieve on the FAST assessment is 18.

The initial part of the assessment focused on the cardiac US, assessing the student’s ability to identify the four chambers of the heart, the pericardium, and the location of fluid accumulation in a pericardial effusion. Students were timed after applying gel to the probe to identify each component.

The second section of the assessment concentrated on the right upper quadrant, examining the students' proficiency in identifying the liver, right kidney, hepatorenal recess, diaphragm, mirror artifact, and the site of fluid accumulation when in a supine position. Likewise, students were timed after applying gel to the probe to identify each component.

The third section focused on the left upper quadrant and assessed the student’s ability to locate the spleen, left kidney, splenorenal recess, and the location where fluid would accumulate in a hemoperitoneum. Again, the students were timed after applying gel to the probe to identify each component.

The final part of the evaluation was the suprapubic region, which evaluated students' ability to identify the bladder and the location where fluid would accumulate. Similarly, students were timed after applying gel to the probe to identify each component.

The US workshop concluded with a post-training participant survey designed to gather feedback on student opinions, experiences, and US competency. The results of the pre-training and post-training surveys were subsequently compared to assess the effectiveness of the workshop. 

## Results

Frequency and percentage distributions were utilized for data analysis, with the data being analyzed using Microsoft Excel (Microsoft Corp., New York, USA). The study collected both qualitative and quantitative data. Surveys were used to gather qualitative data, while student performance in FAST US was assessed using quantitative data.

Pre-testing survey results

In Table 1 for group A, during pre-testing, the tasks where every student (100%) selected the minimum confidence score of one (on a five-point scale) included the correct hand technique for holding the probe, handling the US cable appropriately, using the freeze function on the US, adjusting the US image's depth/zoom, identifying the pouch of Morrison, and locating the bladder trigone. By contrast, group B exhibited a markedly reduced percentage of students choosing the lowest confidence score in pre-testing, with a mere 42.86% scoring one for the task of identifying the bladder trigone, followed by 37.14% for identifying the pouch of Morrison. 

No group A students assigned themselves the highest confidence score of five on the five-point Likert scale for any of the tasks in the pre-testing survey. In contrast, group B contained a noticeable number of students who rated themselves a five for several tasks. Specifically, 17.14% of group B students scored a five for holding the probe in the correct orientation, 8.57% for holding the probe with the appropriate hand technique, 2.86% for correctly handling the cable, 20.0% for using the freeze function, and 5.71% for adjusting depth/zoom, fanning the transducer, identifying the right kidney, and identifying the left kidney. Overall, the survey results indicate that group B students entered the study with higher confidence in their ability to perform a US examination compared to group A, as fewer group B students selected the lowest confidence score for each task and a greater proportion assigned themselves the highest confidence score for a variety of tasks.

**Table 1:** Comparative confidence levels in pre-test FAST ultrasound tasks for groups A and B. Confidence levels were assessed using the Likert scale (1-5), with percentages indicating student responses. *P-value <0.01, **P-value <0.005, ***P-value <0.001. FAST: focused assessment with sonography for trauma, US: ultrasound.

Survey question	Likert scale	Group A	Group B
		Pre-testing (n=33)	Pre-testing (n=38)
Choosing the correct probe to identify a specific landmark/organ	1***	87.87	8.57
	2	12.12	37.14
	3	0	25.71
	4	3.03	28.57
	5	0	0
Holding the probe in its correct orientation	1***	96.97	5.71
	2	3.03	22.86
	3	0	14.29
	4	0	40
	5***	0	17.14
Holding the probe using the proper hand technique	1***	100	5.71
	2	0	34.29
	3	0	25.71
	4	0	25.71
	5***	0	8.57
Manipulating the ultrasound cable correctly while performing the ultrasound	1***	100	8.57
	2	0	34.29
	3	0	22.86
	4	0	31.43
	5***	0	2.86
Applying the aquasonic gel onto the tip of the probe when necessary	1***	87.88	5.71
	2	9.09	25.71
	3	6.06	20
	4	0	28.57
	5	0	20
Using the freeze function on US	1***	100	8.57
	2	0	28.57
	3	0	11.43
	4	0	31.43
	5***	0	20
Altering depth/zoom of the US image	1*	100	31.43
	2	0	28.57
	3	0	22.86
	4	0	11.43
	5*	0	5.71
Fanning/tilting the transducer (US device) to get a better view	1***	96.97	20
	2	3.03	42.86
	3	0	8.57
	4	0	22.86
	5*	0	5.71
Identifying the liver	1**	96.97	28.57
	2	3.03	42.86
	3	0	8.57
	4	0	17.14
	5	0	2.86
Identifying the diaphragm	1*	93.94	28.57
	2	6.06	45.71
	3	0	17.14
	4	0	5.71
	5	0	2.86
Identifying the right kidney	1***	90.91	25.71
	2	9.09	45.71
	3	3.03	11.43
	4	0	11.43
	5*	0	5.71
Identifying the pouch of Morrison	1**	100	37.14
	2	0	34.29
	3	0	14.29
	4	0	11.43
	5	0	2.86
Identifying the four chambers of the heart	1***	90.91	25.71
	2	9.09	45.71
	3	3.03	22.86
	4	0	2.86
	5	0	2.86
Identifying the pericardium	1*	96.97	34.29
	2	3.03	42.86
	3	0	14.29
	4	0	5.71
	5	0	2.86
Identifying the left kidney	1***	93.94	28.57
	2	6.06	42.86
	3	0	14.29
	4	0	8.57
	5*	0	5.71
Identifying the spleen	1***	100	34.29
	2	0	42.86
	3	0	11.43
	4	0	8.57
	5	0	2.86
Identifying the bladder trigone	1*	100	42.86
	2	0	45.71
	3	0	8.57
	4	0	2.86
	5	0	0

Post-testing survey results

In Table 2, the differences between the two groups were less pronounced in the post-testing phase compared to the initial evaluations. However, a larger proportion of students in group B chose the lowest confidence rating (one out of five) than in group A for specific tasks. These tasks included appropriate probe selection, proper probe handling, applying gel to the probe tip, and identifying anatomical structures such as the liver, diaphragm, right kidney, and left kidney. Nevertheless, group A surpassed group B in confidence concerning the task of modifying the depth/zoom of the US image.

**Table 2:** Comparative confidence levels in post-test FAST ultrasound tasks for groups A and B. Confidence levels were assessed using the Likert scale (1-5), with percentages indicating student responses. *P-value <0.01, **P-value <0.005, ***P-value <0.001. FAST: focused assessment with sonography for trauma, US: ultrasound.

Survey question	Likert scale	Group A	Group B
		Post-testing (n=33)	Post-testing (n=38)
Choosing the correct probe to identify a specific landmark/organ	1*	0	7.69
	2	9.09	2.56
	3	9.09	17.95
	4	21.21	20.51
	5	60.61	51.28
Holding the probe in its correct orientation	1*	0	7.69
	2	3.03	0
	3	12.12	15.38
	4	21.21	17.95
	5	63.64	58.97
Holding the probe using the proper hand technique	1	0	5.13
	2	6.06	2.56
	3	15.15	15.38
	4	27.27	20.51
	5	51.52	56.41
Manipulating the ultrasound cable correctly while performing the ultrasound	1	0	5.13
	2	3.03	5.13
	3	15.15	15.38
	4	27.27	20.51
	5	54.55	53.85
Applying the aquasonic gel onto the tip of the probe when necessary	1*	0	7.69
	2	3.03	0
	3	12.12	17.95
	4	12.12	5.13
	5	72.73	69.23
Using the freeze function on US	1	3.03	7.69
	2	0	0
	3	12.12	15.38
	4	21.21	5.13
	5	63.64	71.79
Altering depth/zoom of the US image	1***	30.30	7.69
	2	15.15	12.82
	3	24.24	23.08
	4	21.21	25.64
	5***	9.09	30.77
Fanning/tilting the transducer (US device) to get a better view	1	0	5.13
	2	9.09	2.56
	3	9.09	28.21
	4	27.27	20.51
	5*	54.55	43.59
Identifying the liver	1*	0	7.69
	2	6.06	5.13
	3	15.15	15.38
	4	33.33	23.08
	5	45.45	48.72
Identifying the diaphragm	1*	0	7.69
	2	6.06	5.13
	3	18.18	15.38
	4	21.21	25.64
	5	54.54	46.15
Identifying the right kidney	1*	0	7.69
	2	6.06	0
	3	12.12	17.95
	4	21.21	20.51
	5*	60.61	53.85
Identifying the pouch of Morrison	1	9.09	7.69
	2	6.06	2.56
	3	12.12	17.95
	4	39.39	23.08
	5***	33.33	48.72
Identifying the four chambers of the heart	1	3.03	2.56
	2	6.06	12.82
	3	21.21	17.95
	4	48.48	28.21
	5***	21.21	38.46
Identifying the pericardium	1	3.03	5.13
	2	0	2.56
	3	33.33	20.51
	4	39.39	20.51
	5***	24.24	51.28
Identifying the left kidney	1*	0	7.69
	2	12.12	2.56
	3	6.06	12.82
	4	39.39	25.64
	5*	42.42	51.28
Identifying the spleen	1	3.03	7.69
	2	9.09	2.56
	3	9.09	17.95
	4	39.39	28.21
	5	39.39	43.59
Identifying the bladder trigone	1	6.06	2.56
	2	6.06	5.13
	3	15.15	15.38
	4	21.21	23.08
	5	51.52	53.85

Despite the slight convergence in confidence levels (five out of five) between group A and group B students in the post-testing phase, group B still manifested markedly higher overall self-assurance in their ability to perform a FAST examination. This was evident with significant p-values (p-value <0.001-0.01) across multiple tasks. Specifically, group B students demonstrated elevated confidence in altering the depth of the US image, sweeping the US device, correctly identifying the right and left kidneys, pinpointing the pouch of Morrison, and successfully recognizing the four chambers of the heart as well as the pericardium. To summarize, in the post-testing phase, group B students seemed to display greater confidence across a wider range of tasks compared to group A students. However, it remains challenging to draw definitive conclusions as confidence levels varied depending on the specific task at hand.

Pre-testing assessment results

The assessment calculated the percentage of students who successfully completed each task in the four components of the exam. Additionally, average scores and completion times were determined for each component before and after training, as shown in Tables 3-5. Positive differences indicate that group B had more successful students than group A, while negative differences indicate the opposite. Furthermore, Tables 4-6 compare the average total scores and completion times between groups A and B.

As illustrated in Table 3, group B, before undergoing training, either paralleled or exceeded the performance of group A in successfully executing each task associated with the FAST US examination. The differences between the groups were particularly significant for proper probe selection and technique in the four components of the exam (p<0.005-p<0.01). The smallest difference observed across all components for proper probe selection and technique was 9, indicating that at least nine more students in group B successfully completed that task compared to group A. Other notable differences where group B performed significantly better than group A were in identifying the liver, right kidney, hepatorenal recess, fluid accumulation in a supine position, and left kidney (p<0.001-p<0.01). The differences for these tasks ranged between eight and 12 students.

**Table 3:** Illustration of the number of students in groups A (n=33) and B (n=38) who successfully identified each item under timed conditions prior to training in FAST US (% of students). *P-value <0.01, **P-value <0.005, and ***P-value <0.001. FAST: focused assessment with sonography for trauma, US: ultrasound.

	Cardiac US (group A)	Cardiac US (group B)	Difference
Proper probe selection, technique and standing on the right side of the patient	3 (9.09%)	14 (38.84%)	11*
Able to identify all four chambers of the heart	2 (6.06%)	2 (5.26%)	0
Able to identify pericardium	2 (6.06%)	4 (10.53%)	2
Able to point out where fluid would accumulate in a pericardial effusion	3 (9.09%)	9 (23.68%)	6
Average score for cardiac US (maximum=4)	0.36	0.76	0.40
Average time (sec) to complete cardiac component of FAST US	111.70 s	56.76 s	−54.94 s***
	Right upper quadrant US (group A)	Right upper quadrant US (group B)	Difference
Proper probe selection, technique and standing on the right side of the patient	4 (12.12%)	17 (44.74%)	13**
Able to identify the liver	1 (3.03%)	9 (23.68%)	8*
Able to identify the right kidney	1 (3.03%)	13 (34.21%)	12**
Able to identify hepatorenal recess	0 (0%)	12 (31.58%)	12***
Able to locate diaphragm and mirror artifact (right lung)	0 (0%)	0 (0%)	0
Able to point out where fluid would accumulate when supine	0 (0%)	12 (31.58)	12***
Average score of right upper quadrant (maximum=6)	0.18	1.66	1.48***
Average time (sec) to complete right upper quadrant component of FAST US	117.15 s	59.66 s	−47.49 s***
	Left upper quadrant US (group A)	Left upper quadrant US (group B)	Difference
Proper probe selection, technique and standing on the right side of the patient	3 (9.09.%)	13 (34.21)	10*
Able to identify the spleen	3 (9.09%)	4 (10.53)	1
Able to identify the left kidney	1 (3.03%)	13 (34.21)	12***
Able to identify splenorenal recess (border between spleen and kidney)	0 (0%)	3 (7.89)	3
Able to point out where fluid would accumulate in a hemoperitoneum	0 (0%)	3 (7.89)	3
Average score of the left upper quadrant (maximum=5)	0.24	0.95	0.71***
Average time (sec) to complete left upper quadrant component of FAST US	114.03 s	55.18 s	−58.8 s***
	Suprapubic region US (group A)	Suprapubic region US (group B)	Difference
Proper probe selection, technique and standing on the right side of the patient	7 (21.21%)	20 (52.63%)	13*
Able to identify bladder	4 (12.12%)	7 (18.42%)	3
Able to point out where fluid would accumulate	0 (0%)	2 (5.26%)	2
Average score of the left upper quadrant (maximum=3)	0.42	0.76	0.34*
Average time (sec) to complete suprapubic region component of FAST US	79.52 s	30.32 s	−49.2 s**

As illustrated in Table 4, group B achieved a statistically significant higher average total score (p<0.001), exceeding group A's performance by 3.10 points. In addition to this, the students in group B were more efficient, completing the FAST exam faster by a notable 220.47 seconds (p<0.001) compared to their group A counterparts. These metrics provide substantial evidence affirming that group B held a performance advantage over group A even prior to receiving training in FAST US.

**Table 4:** Illustration of the average results from students in groups A (n=33) and B (n=38) prior to training in FAST US. *P-value <0.01, **P-value <0.005, ***P-value <0.001. FAST: focused assessment with sonography for trauma, US: ultrasound.

	Group A	Group B	Difference
Average total score (maximum=18)	1.03	4.13	3.10***
Average time (sec) to complete FAST US exam	422.39 s	201.92 s	−220.47 s***

Post-testing assessment results

As depicted in Table 5, the outcomes following the training closely mirrored those seen prior to the training intervention. Group B continued to outpace group A in successfully executing each task spanning the four components of the exam. Once again, the probe selection and technique task favored group B, with differences ranging from 11 to 16 students (p<0.001-p<0.01). Interestingly, the post-training differences in the left upper quadrant and suprapubic region tasks ranged from 11 to 19 students (p<0.001-p<0.01), indicating group B's proficiency in these areas. Notably, group B excelled in other specific domains, such as identifying where to locate fluid accumulation in pericardial effusion and when supine, with differences of 22 and 16 students, respectively (p<0.001).

As reflected in Table 6, group B not only achieved a higher average total score but also a quicker mean completion time. With a statistically significant difference of 4.01 (p<0.001) in the average total score and a completion time advantage of 50.24 seconds (p<0.001), it's clear that more students in group B finished the FAST US examination successfully and more rapidly than their counterparts in group A.

**Table 5:** Illustration of the number of students in groups A (n=33) and B (n=38) who successfully identified each item under timed conditions after training in FAST US (% of students). *P-value <0.01, **P-value <0.005, ***P-value <0.001. FAST: focused assessment with sonography for trauma, US: ultrasound.

	Cardiac US (group A)	Cardiac US (group B)	Difference
Proper probe selection, technique and standing on the right side of the patient	23 (69.69%)	37 (97.37%)	14**
Able to identify all four chambers of the heart	15 (45.45%)	17 (44.74%)	2
Able to identify pericardium	12 (36.36%)	18 (47.37%)	6
Able to point out where fluid would accumulate in a pericardial effusion	12 (36.36%)	34 (89.47%)	22***
Average score for cardiac US (maximum=4)	1.91	2.79	0.88**
Average time (sec) to complete cardiac component of FAST US	67.85 s	48.87 s	−18.98 s***
	Right upper quadrant US (group A)	Right upper quadrant US (group B)	Difference
Proper probe selection, technique and standing on the right side of the patient	20 (60.61%)	36 (94.74%)	16***
Able to identify the liver	25 (75.76%)	30 (78.95%)	5
Able to identify the right kidney	29 (87.88%)	32 (84.21%)	3
Able to identify hepatorenal recess	25 (75.76%)	34 (89.47%)	9
Able to locate diaphragm and mirror artifact (right lung)	26 (78.79%)	29 (76.32%)	3
Able to point out where fluid would accumulate when supine	20 (60.61%)	36 (94.74%)	16***
Average score of right upper quadrant (maximum=6)	4.42	5.18	0.76*
Average time (sec) to complete right upper quadrant component of FAST US	60.79 s	47.55 s	−13.24 s**
	Left upper quadrant US (group A)	Left upper quadrant US (group B)	Difference
Proper probe selection, technique and standing on the right side of the patient	22 (66.66%)	35 (92.11%)	13*
Able to identify the spleen	16 (48.48%)	30 (78.95%)	14*
Able to identify the left kidney	23 (69.70%)	35 (92.11%)	12
Able to identify splenorenal recess (border between spleen and kidney)	18 (54.55%)	33 (86.84%)	15**
Able to point out where fluid would accumulate in a hemoperitoneum	12 (36.36%)	31 (81.58%)	19***
Average score of the left upper quadrant (maximum=5)	2.79	4.32	1.53***
Average time (sec) to complete left upper quadrant component of FAST US	57.27 s	46.16 s	−11.11 s*
	Suprapubic region US (group A)	Suprapubic region US (group B)	Difference
Proper probe selection, technique and standing on the right side of the patient	25 (75.76%)	37 (97.36%)	13*
Able to identify bladder	18 (54.55%)	29 (76.32%)	11
Able to point out where fluid would accumulate	20 (60.61%)	32 (84.21%)	12*
Average score of the left upper quadrant (maximum=3)	1.91	2.55	0.64***
Average time (sec) to complete suprapubic region component of FAST US	27.67 s	20.76 s	−6.91 s***

**Table 6:** Illustration of the average results from students in groups A (n=33) and B (n=38) after training in FAST US. *P-value <0.01, **P-value <0.005, and ***P-value <0.001. FAST: focused assessment with sonography for trauma, US: ultrasound.

	Group A	Group B	Difference
Average total score (maximum=18)	10.88	14.89	4.01***
Average time (sec) to complete FAST US exam	213.58 s	163.34 s	−50.24 s***

## Discussion

The core objective of this educational intervention was to appraise the efficacy of FAST US training in bolstering the acquisition and practical application of this indispensable skill among medical students. Emerging literature underscores the significance of early integration of US education within medical school curricula, residency, and fellowship training, as evidenced by improved performance on image interpretation tests. This accentuates the urgency of identifying the optimal timing for introducing FAST US training [9-11]. The FAST US examination holds pivotal importance in emergency scenarios, rendering its training invaluable for medical professionals and indispensable for delivering efficient patient care. By employing the FAST examination, healthcare providers can swiftly detect fluid accumulation in critical regions such as the pericardial, pleural, and intraperitoneal spaces-often comprising blood in trauma cases. This underscores the criticality of the FAST examination in promptly identifying life-threatening conditions such as hemoperitoneum in patients with abdominal injuries.

The inherent benefits of FAST encompass its non-invasive nature and absence of ionizing radiation, making it a viable alternative to X-ray scans [12]. Particularly in emergency departments and trauma centers, FAST exhibits advantages over computed tomography scans, offering cost-effectiveness, speed, reduced complications, and shorter patient stays [13,14]. Moreover, FAST examinations can be conducted immediately upon a patient's arrival, eliminating the need for transportation to imaging rooms. A randomized controlled clinical trial in 2015 demonstrated that US usage can decrease the demand for CT scans by half [15]. Further efficiency gains are evident in diagnoses time, with studies showcasing a mean time of 53 minutes when using FAST, as opposed to 151 minutes without [16].

An illustrative example of early US education integration comes from the University of California, Irvine, which began incorporating US education into its four-year medical degree curriculum [17]. This initiative establishes guidelines for incorporating US methods into physical examinations, emphasizing the advantages of early US training for medical students in terms of diagnostic precision and procedural prowess. A Swiss study evaluated the impact of ultrasound instructions at different stages of clinical training on medical students' competency in interpreting and applying POCUS procedures [7]. Results highlighted the feasibility of students acquiring essential skills for POCUS interpretation and application, with late clinical students excelling in written assessments. Nevertheless, clinical skills exhibited no significant variation between early and late clinical students.

While FAST exams offer portability, they are not without limitations. Effectiveness hinges on the provider's proficiency, necessitating specific training in FAST beyond general US skills. Moreover, the reliability of FAST is patient-dependent and influenced by factors such as BMI and subcutaneous emphysema [18].

The study's compelling data reveal pronounced differences between medical students with prior US experience (group B) and those without (group A) following training. These disparities are attributed to various factors. Importantly, substantial variations in students' confidence levels, as evidenced by pre- and post-training surveys, underscore the positive impact of FAST US training. Group B exhibited greater confidence enhancement despite a lack of prior US experience. This suggests that early US training can boost confidence in performing FAST US exams.

Specific challenges were evident for both groups. Group B displayed superior performance in the right upper quadrant, attributable to prior US experience. However, challenges persisted in areas such as the left upper quadrant, left spleen, splenorenal recess, and identifying fluid accumulation in the hemoperitoneum. These challenges underscore the need for targeted training modules focusing on these areas during FAST examination education. Both groups stand to benefit from such modules to enhance accuracy and proficiency.

During the post-training phase, group B sustained their exceptional performance, replicating their pre-training proficiency. Their mastery of probe selection, technique, and tasks related to the left upper quadrant and suprapubic region underscores advanced proficiency in these aspects. Additionally, group B's competence in identifying fluid accumulation highlights the benefits of prior US experience in augmenting performance.

Moreover, group B demonstrated superior average total scores and completion times, indicative of the positive impact of prior US experience on performance and efficiency. This highlights the advantages of incorporating FAST training early in medical school curricula, enabling students to cultivate proficiency and efficiency in conducting FAST exams. Collectively, these findings reinforce the imperative of timely FAST training for students.

To seamlessly integrate FAST training, medical schools should consider key recommendations. Training should be introduced after students have developed a fundamental grasp of anatomy and US principles, allowing them to build on their anatomical foundation while honing their US skills. A multifaceted approach involving didactic sessions, hands-on practice, and simulation-based training is pivotal [19]. Didactic sessions should cover US physics principles, relevant anatomy, and image interpretation. Hands-on practice, guided by experienced instructors, enables the practical application of knowledge. Simulation-based training, utilizing high-fidelity US simulators or standardized patients, further enhances skills and confidence.

Adequate resources and support are vital, encompassing access to US equipment, training materials, and mentorship from skilled faculty members or sonographers. Integrating training into clinical rotations, particularly in emergency medicine or trauma specialties, provides exposure to real-world scenarios where skills can be practically applied [20].

Despite the consensus on the importance of integrating FAST training into medical school curricula, several obstacles must be addressed for successful implementation [21]. A survey of administrators from 82 U.S. Medical Schools revealed a high level of agreement (78.9%) regarding US training incorporation, alongside notable challenges hindering its integration [22]. Limited curriculum space and inadequate financial support pose barriers to FAST training integration. Addressing these challenges could involve training a subset of medical students to proficiently teach their peers. While this approach may not reach all students, it could help bridge training gaps and facilitate integration [23]. Overcoming these hurdles is pivotal for the successful implementation of FAST training and its benefits in medical education.

Limitations

To grasp the full scope of the study, it is crucial to recognize several limitations. The study did not include detailed information about the participants' extent and duration of prior US training, hindering our understanding of their US knowledge depth and its influence on the results.

A further limitation includes the simplistic binary interpretation of the Likert scale in the survey, possibly overlooking nuanced improvements or challenges. The study did not take into account the varying learning styles of students, such as Kolb's four categories: divergent, assimilative, convergent, and accommodative. While many medical students (45.9%) tend to learn primarily through observation, as assimilators, the unique needs of other learning styles may have been neglected, potentially affecting the overall success of the training program [24,25].

Another constraint was the absence of evaluation of participants' anatomical knowledge, particularly related to structures relevant to the FAST examination. This omission might have impacted comprehension of performance influence. Moreover, the study focused on immediate proficiency without investigating the long-term retention of FAST skills. Lastly, the exclusive focus on the FAST examination method neglects other US examination methods, like POCUS and e-FAST, used in practice.

Future research

The limitations identified in this study emphasize the need for future research to enhance understanding of the long-term impact and effectiveness of US training programs. Future studies should aim to gather more detailed data on participants' previous US training, allowing a deeper analysis of their capabilities.

There is also a need to explore the interpretation of assessment scales in greater detail, considering all values instead of only the most extreme. Understanding students' different learning styles and their unique needs would provide further insights into the training program's success.

Investigating participants' anatomical knowledge, especially as it pertains to the FAST examination, will clarify how performance may have been influenced. Future work must also delve into the long-term retention of skills to gauge the training's ongoing effectiveness. Finally, future research should incorporate additional US examination methods, offering a more comprehensive assessment and a broader understanding of participants' proficiency.

## Conclusions

This study emphasizes the critical importance of previous US experience in acquiring and applying FAST US training among medical students, going beyond traditional views on medical education. It reveals a complex relationship between prior experience, training efficiency, and clinical expertise, making a strong case for integrating FAST US training into medical curricula. The research highlights how prior US experience significantly boosts the learning curve, confidence, and performance in FAST examinations (p<0.01). Students with such experience not only learn faster but also perform with higher accuracy, indicating that this expertise can improve patient care efficiency and urgency.

Furthermore, the paper unveils uncharted areas, showing how prior US experience can revolutionize medical education. It provides practical insights for medical schools to foster a generation of healthcare providers ready for real-world emergencies with increased competence and assurance. The study also emphasizes the significance of FAST in emergency and trauma care, characterized by speed, cost-efficiency, and patient-friendliness. It advocates a paradigm shift in medical education, aligning with modern healthcare challenges and technology.

The study also emphasizes the significance of FAST in emergency and trauma care, which is characterized by speed, cost-efficiency, and patient-friendliness. It advocates a paradigm shift in medical education, aligning with modern healthcare challenges and technology. In conclusion, the integration of FAST training, tailored to students' prior US experience, is essential for equipping future healthcare professionals with the skills needed for emergencies, improving patient care and outcomes. The findings highlight the importance of early integration of FAST training within medical education to enhance skill acquisition and patient care.
